# Curcumin Protects Microglia and Primary Rat Cortical Neurons against HIV-1 gp120-Mediated Inflammation and Apoptosis

**DOI:** 10.1371/journal.pone.0070565

**Published:** 2013-08-06

**Authors:** Luyan Guo, Yanyan Xing, Rui Pan, Mingliang Jiang, Zheng Gong, Liqing Lin, Junbing Wang, Guoyin Xiong, Jun Dong

**Affiliations:** 1 Department of Gynaecology and Obstetrics, the First Affiliated Hospital of Sun Yat-sen University, Guangzhou, Guangdong Province, China; 2 Department of Pathophysiology, Key Laboratory of the State Administration of Traditional Chinese Medicine, Medical College of Jinan University, Guangzhou, Guangdong Province, China; 3 Institute of Brain Research, Medical College of Jinan University, Guangzhou, Guangdong Province, China; 4 Guangdong-Hongkong-Macau Institute of CNS Regeneration (GHMICR), Jinan University, Guangzhou, Guangdong Province, China; 5 Department of Orthopedics, the First Affiliated Hospital, Medical College of Jinan University, Guangzhou, Guangdong Province, China; University of South Alabama, United States of America

## Abstract

Curcumin is a molecule found in turmeric root that has anti-inflammatory, antioxidant, and anti-tumor properties and has been widely used as both an herbal drug and a food additive to treat or prevent neurodegenerative diseases. To explore whether curcumin is able to ameliorate HIV-1-associated neurotoxicity, we treated a murine microglial cell line (N9) and primary rat cortical neurons with curcumin in the presence or absence of neurotoxic HIV-1 gp120 (V3 loop) protein. We found that HIV-1 gp120 profoundly induced N9 cells to produce reactive oxygen species (ROS), tumor necrosis factor-α (TNF-α) and monocyte chemoattractant protein-1 (MCP-1). HIV-1 gp120 also induced apoptosis of primary rat cortical neurons. Curcumin exerted a powerful inhibitory effect against HIV-1 gp120-induced neuronal damage, reducing the production of ROS, TNF-α and MCP-1 by N9 cells and inhibiting apoptosis of primary rat cortical neurons. Curcumin may exert its biological activities through inhibition of the delayed rectification and transient outward potassium (K^+^) current, as curcumin effectively reduced HIV-1 gp120-mediated elevation of the delayed rectification and transient outward K^+^ channel current in neurons. We conclude that HIV-1 gp120 increases ROS, TNF-α and MCP-1 production in microglia, and induces cortical neuron apoptosis by affecting the delayed rectification and transient outward K^+^ channel current. Curcumin reduces production of ROS and inflammatory mediators in HIV-1-gp120-stimulated microglia, and protects cortical neurons against HIV-1-mediated apoptosis, most likely through inhibition of HIV-1 gp120-induced elevation of the delayed rectification and transient outward K^+^ current.

## Introduction

The human immunodeficiency virus type 1 (HIV-1) pandemic has claimed over 20 million lives, with 38.6 million people worldwide currently infected (2009 AIDS Epidemic Update by UNAIDS/WHO, www.unaids.org), and will continue to contribute to human morbidity and mortality as there is no vaccine available. HIV-1-associated neurological disorders such as neurodegenerative diseases or neurocognitive disorders are common neurological complications in patients chronically infected with HIV-1. Prior to antiretroviral therapy (ART), neurologic disorders were the first manifestation of symptomatic HIV-1 infection, affecting roughly 10% –20% of patients and up to 60% of patients in the advanced stages of acquired immunodeficiency syndrome (AIDS) [1]. Neuroinflammation and microglial overactivation are involved in the pathogenesis of HIV-1-associated neurologic disorders. Microglia are the major inflammatory cells in the central nervous system (CNS). Over-activated microglial cells cause overproduction of various proinflammatory cytokines including tumor necrosis factor-alpha (TNF-α), interleukin 1β (IL-1β) and chemokines, which are believed to contribute to HIV-1-associated neurologic disorders [2].

Uniquely, HIV-1 causes neuronal injury, cell loss and dysfunction in the CNS through soluble virus proteins rather than productive virus infection, because there is no evidence that HIV-1 directly infects neurons. One of HIV-1 proteins, gp120, has been shown to directly interact with neurons, leading to neuronal apoptosis [3]. In fact, the severity of brain damage is positively correlated with level of gp120 expression in the brain of HIV-1-infected individuals [2]. These findings provide evidence that gp120 plays a key role in HIV-1-associated nervous system impairment. Recent studies have demonstrated that neuronal voltage-gated potassium (Kv) channels are involved in HIV-1-associated neuronal injury [4]. HIV-1 gp120 enhances A-type transient outward K^+^ currents (I_A_) in neurons, resulting in neuronal apoptosis [4]. Inhibition of the gp120- induced increase of I_A_ attenuates gp120-mediated apoptosis of neurons [4], and improves learning and memory in animals as well [5], [6]. Actually, overactivation of voltage-gated Kv channels can trigger excessive K^+^ efflux and intracellular K^+^ depletion, which are early ionic events in apoptotic cascades of neurons [7]. These data are consistent with early findings that K^+^ channel-mediated K^+^ efflux contributes to ischemia-triggered apoptosis of neurons, and that the K^+^ channel blocker tetraethylammonium (TEA) attenuates hypoxia- and ischemia-induced neuronal death in vitro and in vivo [8]. Thus, HIV-1 gp120 can directly cause neuronal injury by enhancing I_A_ in neurons.

Curcumin is a hydrophobic polyphenol derived from the rhizomatous herbaceous species *Curcuma longa*, which belongs to the Zingiberaceae family. Curcumin exhibits a wide variety of antioxidant [9], anti-inflammatory [10], antimicrobial [11], and anticarcinogenic activities [12]. In a previous study, we demonstrated that curcumin could improve learning and memory abilities in rats with HIV-1 gp120-induced memory disorders [13]. This study was undertaken to determine whether curcumin has a protective effect against HIV-1 gp120-mediated neurotoxicity by reducing microglial inflammation and by preventing neuronal apoptosis through acting on neuronal voltage-gated K^+^ channels.

## Materials and Methods

### HIV-1 gp120 V3 Loop Peptide, Curcumin and Other Reagents

The HIV-1 IIIB gp120 V3 loop was synthesized by the Shanghai Apeptide (Shanghai, China; sequence: Asn-Asn-Thr-Arg-Lys-Ser-Ile-Arg-Ile-Gln- Arg-Gly-Pro -Gly-Arg-Ala-Phe-Val-Thr-Ile-Gly-Lys-Ile-Gly; molecular formula: C_114_H_199_N_41_O_31_; molecular weight: 2,640.06). Curcumin and N-acetylcysteine (NAC, a potent antioxidant) were purchased from Sigma Aldrich-Fluka (St. Louis, MO, USA). Tetraethylammonium bromide (TEA) and 4-aminopyridine (4-AP), two selective blockers of voltage-gated K^+^ currents, were procured from Aladdin (Shanghai, China) and Johnson Matthey-Alfa Aesar (London, United Kingdom), respectively.

### Animals

A total of 15 one-day-old Sprague-Dawley rats were supplied by the Animal Experimental Center of the Southern Medical University of China [License No. SCXK (yue) 2006-0015]. All animal use procedures were reviewed and approved by the Medical Ethics Committee of Jinan University.

### Cell Cultures

The N9 murine microglial cell line was kindly provided by Dr. Yun Bai (Department of Medical Genetics, Third Military Medical University, ChongQing, China) [14], and cultured in Dulbecco’s Modified Eagle Medium (DMEM)/F12 supplemented with 10% fetal bovine serum (FBS) (Thermo Scientific Hyclone, Logan, UT, USA), 2 mM glutamine, 100 U/ml penicillin and 100 µg/ml streptomycin at 37°C in a 5% CO_2_ humidified incubator.

Primary rat cortical neurons were prepared and cultured using a protocol previously described [15], with a slight modification. Briefly, 1-day-old neonatal Sprague-Dawley rats were sacrificed and sterilized with 75% alcohol. Their scalps and skulls were incised to expose both cerebral hemispheres that were then harvested. The cerebral cortex was washed twice with ice-cold D-Hanks’ solution, and the blood vessels were removed with straight iris forceps. Fresh cortical tissues were sectioned into approximately 1-mm slices using forceps and then placed in a 15-mL centrifuge tube containing D-Hanks’ solution and 0.25% trypsin (volume ratio = 2∶1). The sectioned tissues were digested for 10 min at 37°C, and the digestion was terminated using 10% FBS/DMEM/F12. The digested tissues were then mechanically dispersed by lightly blowing on them with a pipette. The solution was centrifuged for 5 min at 1,000 rpm, and the supernatant was discarded. The cells were subsequently resuspended in 10% FBS/DMEM/F12, and the cell suspension was filtered into a new 15-mL centrifuge tube through a 200 mesh stainless steel screen cloth. The cell suspension was transferred to a culture flask to allow differential adhesion to occur to remove fibroblasts. Next, neurons were seeded at a density of 1 × 10^6^ cells/well onto poly-L-lysine (0.1 mg/mL, Sigma Aldrich-Fluka, St. Louis, MO, USA)-coated coverslips in a 6-well culture plate, followed by incubation in a saturated, humidified incubator containing 5% CO_2_ and 95% air at 37°C. After 4 h of in vitro incubation, the culture medium was replaced with 2% B27 neurobasal medium (Life Technologies-Gibco, Grand Island, NY, USA). Thereafter, half of the medium was replaced with fresh medium twice a week. The cultures were monitored to ensure that neurons constituted ≥95% of the total cell population. All experiments were performed using primary rat cortical neurons cultured for 7 days.

### Measurement of Cell Viability

Effects of HIV-1 gp120 V3 loop and curcumin on microglia viability were evaluated using the MTT assay. Briefly, N9 microglia (1×10^4^ cells/well) were seeded into a 96-well culture plate. After adherence, the cells were treated with various concentrations of curcumin (1, 5, 10, 15, and 20 µM), HIV-1 gp120 V3 loop peptide (0.5, 1, 2, and 4 µg/mL), or heat-inactivated HIV-1 gp120 V3 loop peptide at 4 µg/mL for 24 h. The medium was removed, and cells were incubated with 100 µL (0.5 mg/mL) of 3-(4,5-Dimethyl-2-Thiazolyl)-2,5-diphenyl tetrazolium bromide (MTT, Sigma Aldrich-Fluka, St. Louis, MO, USA) for 4 h at 37°C. The formazan crystals that developed in the cells were solubilized with dimethyl sulfoxide (DMSO), and the MTT formazan level was determined by measuring the absorbance at 490 nm with a microplate reader (Tecan, Switzerland).

### Measurement of ROS Production in Microglia

ROS production in microglia was measured using fluorescence microscope and flow cytometric analysis (FACS).

To quantitate intracellular ROS production in microglia by a fluorescence microscopy, microglia were seeded at a density of 2 × 10^5^ cells/well in a 6-well culture plate. These cells were pre-incubated with curcumin (15 µM), NAC (3 mM), 4-AP (2 mM), or TEA (2 mM) for 2 h, and inoculated with HIV-1 gp120 V3 loop at various concentrations. After 1 h incubation, cells were washed with PBS. 2′-7′-dichloro-dihydro-fluorescein diacetate (DCFH-DA, 10 mM) diluted 3,000-fold in serum-free medium was added to the cells and then incubated for 40 min at 37°C. Cells were washed three times with PBS to remove extracellular DCFH-DA, and then subjected to image texture analysis using fluorescence microscope (IX-71, Olympus, Japan) to excite the cells at 488 nm and then to measure the emission at 525 nm. The mean fluorescence intensity (MFI) of the ROS was analyzed using Image-Pro Plus 6.0 software.

To measure ROS production in microglia by FACS, cells treated with gp120, curcumin, or both were digested with 0.25% trypsin, and then collected in a centrifuge tube. DCFH-DA (10 mM) diluted 3,000-fold in serum-free medium was used to resuspend the cells, and then incubated for 40 min at 37°C. The cells were washed three times with PBS to remove extracellular DCFH-DA, and were then resuspended in 300 µL of PBS. Cells were subjected to FACS using a FACSCalibur (BD Biosciences, San Jose, CA, USA), and 10,000 cells from each group were acquired to determine MFI. The data were analyzed using CellQuest software, and the MFI of each treatment group was compared with that of control group.

### Quantitative Real-time RT-PCR Analysis

Quantitative real-time RT-PCR (qPCR) analysis was used to measure changes of TNF-α and MCP-1 mRNA abundance in microglia in response to HIV-1 gp120 V3 loop treatment in the presence or absence of curcumin, NAC, 4-AP, or TEA. Briefly, microglia were seeded at a density of 2 × 10^5^ cells/well in a 12-well culture plate. After pre-incubation with curcumin (15 µM), NAC (3 mM), 4-AP (2 mM), or TEA (2 mM) for 2 h, cells were exposed to HIV-1 gp120 V3 loop (1 µg/mL) for 3 h. Total RNA was extracted from cell pellets using TRIzol reagent (Takara, Japan), and the integrity of purified RNA samples was confirmed by electrophoresis on 1% agarose gels. Total RNA (2 µg) was used in a 25 µl first strand cDNA synthesis using a reverse transcription system (Promega, USA). The cDNA (2 µl) was used for qPCR with specific primers for TNF-α, MCP-1 and GAPDH: TNF-α, 5′-GACCCTCACACTC AGATCATCTTCT-3′ (forward) and 5′-CCTCCACTTGGTGGTTTGCT-3′ (reverse); MCP-1, 5′- ATCCCAATGAGTAGGCTGGAGAGC-3′ (forward) and 5′-CAGA AGTG CTTGAGGTGGTTGTG-3′ (reverse); and GAPDH, 5′-GTCTTCACCACCATGGAGA AGGC-3′ (forward) and 5′-ATGCCAGTGAGCTTCCCGTTCAGC-3′ (reverse). The qPCR conditions were 95°C for 30 s, followed by 40 cycles of 95°C for 5 s, 53°C for 30 s, and 72°C for 30 s for TNF-α and GAPDH, and 40 cycles of 95°C for 5 s, 56°C for 30 s, and 72°C for 30 s for MCP-1. The fluorescence intensity of SYBR green was measured automatically during qPCR annealing steps to quantitate mRNA copies of TNF-α and MCP-1.

### Analysis of Cortical Neuron Apoptosis

Microglia were seeded at a density of 2×10^5^ cells/well in a 6-well culture plate. After incubating the cells with curcumin (15 µM), 4-AP (2 mM), or TEA (2 mM) for 2 h, the HIV-1 gp120 V3 loop (1 µg/mL) was added, followed by incubation for 12 h. The supernatant from each well was harvested and used as the microglia- conditioned medium to treat neurons. Briefly, primary rat cortical neurons cultured for 7 days were treated with the corresponding conditioned media for 24 h. Cells were then subjected to apoptosis analysis using Hoechst 33342 staining and Western blotting analysis.

To determine cortical neuron apoptosis by Hoechst 33342 staining, cells were incubated with 10 µg/mL of Hoechst 33342 (Beyotime Institute of Biotechnology, Haimen, Jiangsu Province, China) in serum-free medium for 20 min. After washing 3 times with PBS, cells were subjected to image texture analysis using fluorescence microscope (IX-71, Olympus, Japan) to excite the cells at 350 nm and then to measure the emission at 460 nm. The MFI of the cell nuclei was analyzed using Image-Pro Plus 6.0 software.

To assess cortical neuron apoptosis by Western blotting analysis, total cellular proteins were extracted from cortical neurons using RIPA lysis buffer (25 mM Tris–HCl, pH 7.6, 150 mM NaCl, 1% NP-40, 1% sodium deoxycholate, 0.1% SDS, 1 mM PMSF) from Thermo Fisher Scientific (Waltham, MA, USA). Protein concentration was measured using a BCA protein assay kit (Thermo Fisher Scientific, Waltham, MA, USA). Each protein sample (40 µg) was separated in a 10% SDS- PAGE gel. Following electrophoresis, the separated proteins were electrotransferred to polyvinylidene difluoride membranes (PVDF) and blocked in 5% non-fat dried milk/Tris-buffered saline-Tween 20 (TBST) for 2 h at room temperature. The membranes were incubated with primary antibodies (1∶1,000 diluted rabbit anti-rat cleaved caspase-3 from Abcam, Cambridge, UK, and 1∶2,000 diluted rabbit anti-rat GAPDH from Cell Signaling Technology, Boston, MA, USA) overnight at 4°C. After washing 3 times with TBST, the blots were incubated with a goat anti-rabbit IgG antibody (1∶2,000 dilution, Epitomics, Burlingame, CA, USA) at room temperature for 1 h, and the immunoblots were detected using an ECL kit (Millipore, Billerica, MA, USA). Quantity One software (Bio-Rad, Hercules, CA, USA) was employed to quantify the immunoreactive bands. The ratio of cleaved caspase-3 versus GAPDH was then determined.

### Whole-cell Patch Clamp Recordings of Neuronal Voltage-gated Potassium Channels

Whole-cell patch clamp recordings of rat cortical neuron bioactivity were performed in 35-mm culture dishes on an inverted microscope stage (Olympus) using an Axopatch 200B amplifier (Axon, USA). Patch electrodes were created with a P-97 micropipette puller (Sutter, USA) using a 3–5 MΩ tip resistance. The electrodes were advanced toward the cells using an MP-285 micromanipulator (Sutter, USA). We chose neurons with a plump soma and an obvious halo for the recordings. After establishing a whole-cell patch configuration, the cells were allowed to stabilize for 3–5 min prior to testing. The recorded cells were maintained at –80 mV during voltage clamping. Whole-cell outward K^+^ currents were induced through voltage steps ranging from −60 mV to +60 mV in 10-mV increments. A different extracellular solution was employed when recording the delayed rectifier K^+^ current and the transient A-type K^+^ current (I_A_). First, we recorded K^+^ current without chemical agents present in the bath solution. Individual reagents were then applied alone or in combination in a 5-min step perfusion, followed by a 5-min reagent wash-out. The reagents applied in these assays included HIV-1 gp120 V3 loop alone, HIV-1 gp120 V3 loop plus curcumin, and curcumin alone. The final concentrations of HIV-1 gp120 V3 loop and curcumin in the perfusion solution were 5 µg/mL and 15 µM, respectively. After incubation of neurons with HIV-1 gp120 V3 loop, curcumin, or both for 5 min, cellular potassium current was recorded. The reagents were then washed out, and K^+^ current was recorded again. The current signals were filtered at 1 kHz and digitized at 5 kHz using a Digidata 1320A digitizer (Axon, USA). The current and voltage traces were displayed and recorded using the pCLAMP 10.2 data acquisition/analysis system.

The pipette solution consisted of 100 mM L-potassium aspartate, 40 mM KCl, 1 mM MgCl_2_, 10 mM EGTA, 10 mM HEPES and 2 mM Na_2_-ATP, buffered to a pH of 7.2 with KOH. The extracellular solution used to record the delayed rectifier K^+^ current contained 125 mM NaCl, 5 mM KCl, 1 mM MgCl_2_, 1 mM CoCl_2_, 5 mM glucose, 10 mM HEPES, 20 mM sucrose, 5 mM 4-AP and 1 µM tetrodotoxin (TTX, a sodium channel blocker, Hebei Fisheries Research Institute). The extracellular solution employed to record the I_A_ current contained 125 mM NaCl, 5 mM KCl, 1 mM MgCl_2_, 1 mM CoCl_2_, 5 mM glucose, 10 mM HEPES, 20 mM sucrose, 25 mM TEA and 1 µM TTX. Both types of extracellular solutions were buffered to a pH of 7.4 with NaOH and adjusted to 280–300 mOsm with sucrose. All experiments were performed at room temperature (22–23°C), and repeated 6 times.

### Statistics

All data were normally distributed and presented as the mean ± standard deviation (mean ± SD). In cases of multiple comparisons, data were analyzed using a one-way analysis of variance (one-way ANOVA). A *p* value of less than 0.05 was considered statistically significant.

## Results

### Effects of HIV-1 gp120 V3 Loop and Curcumin on Microglial Viability

**Figure 1:**
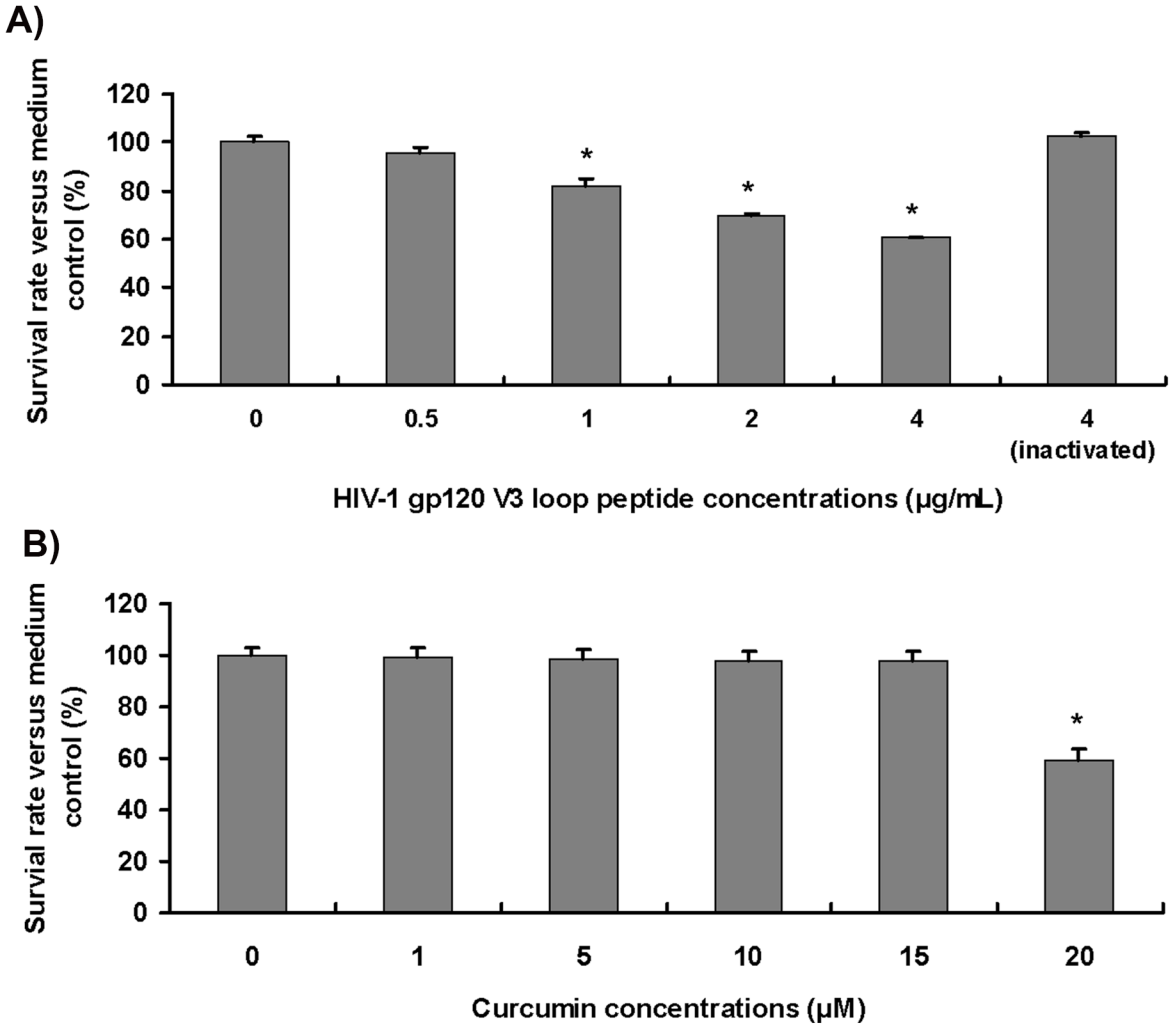
Effects of HIV-1 gp120 V3 loop and curcumin on microglial viability. Microglial cells (mouse N9 cell line) were treated with HIV-1 gp120 V3 loop at various concentrations (0.5, 1, 2, and 4 µg/mL), curcumin at various concentrations (1, 5, 10, 15 and 20 µM), or 4 µg/mL of heat-inactivated HIV-1 gp120 V3 loop for 24 h. Cells were subjected to the MTT assay for viability analysis. A) and B) show the effects of HIV-1 gp120 V3 loop and curcumin on microglial viability, respectively. *, *p*<0.05: experimental group versus medium control. Experiments were repeated 9 times. Inactivated: 4 µg/mL of heat-inactivated HIV-1 gp120 V3 loop.

We used the MTT assay to study the effects of HIV-1 gp120 V3 loop and curcumin on microglial viability. We found that HIV-1 gp120 V3 loop reduced microglia viability in a dose-dependent manner. Compared with medium control (100% of microglial viability), HIV-1 gp120 V3 loop at 1, 2 and 4 µg/mL significantly reduced microglial viability to 82.1±3.0%, 69.5±1.1% and 60.5±0.6%, respectively, whereas HIV-1 gp120 V3 loop at 0.5 µg/mL or lower concentrations, and heat-inactivated HIV-1 gp120 V3 loop at 4 µg/mL had no effect on cell viability (Fig. 1A). Because we wanted to mimic the slow progress and long continuance of HIV-1-related neurologic disorders, we chose gp120 V3 loop at 1 µg/mL to treat microglia for investigating HIV-1-associated neurotoxicity as this concentration of HIV-1 gp120 V3 loop is the lowest dose in inducing a significant reduction of microglial viability.

The MTT assay was also employed to determine the effect of curcumin on microglial viability. Compared with the medium control, curcumin at 15 µM or lower concentrations had no toxic effects on microglial viability (Fig. 1B), whereas curcumin at 20 µM significantly reduced microglial viability to 59.6±4.1% (Fig. 1B). Thus, curcumin at 15 µM was chosen as the experimental concentration in the studies of anti-HIV-1 gp120 neurotoxicity.

### Curcumin Attenuated HIV-1 gp120-mediated ROS Production in Microglia

**Figure 2:**
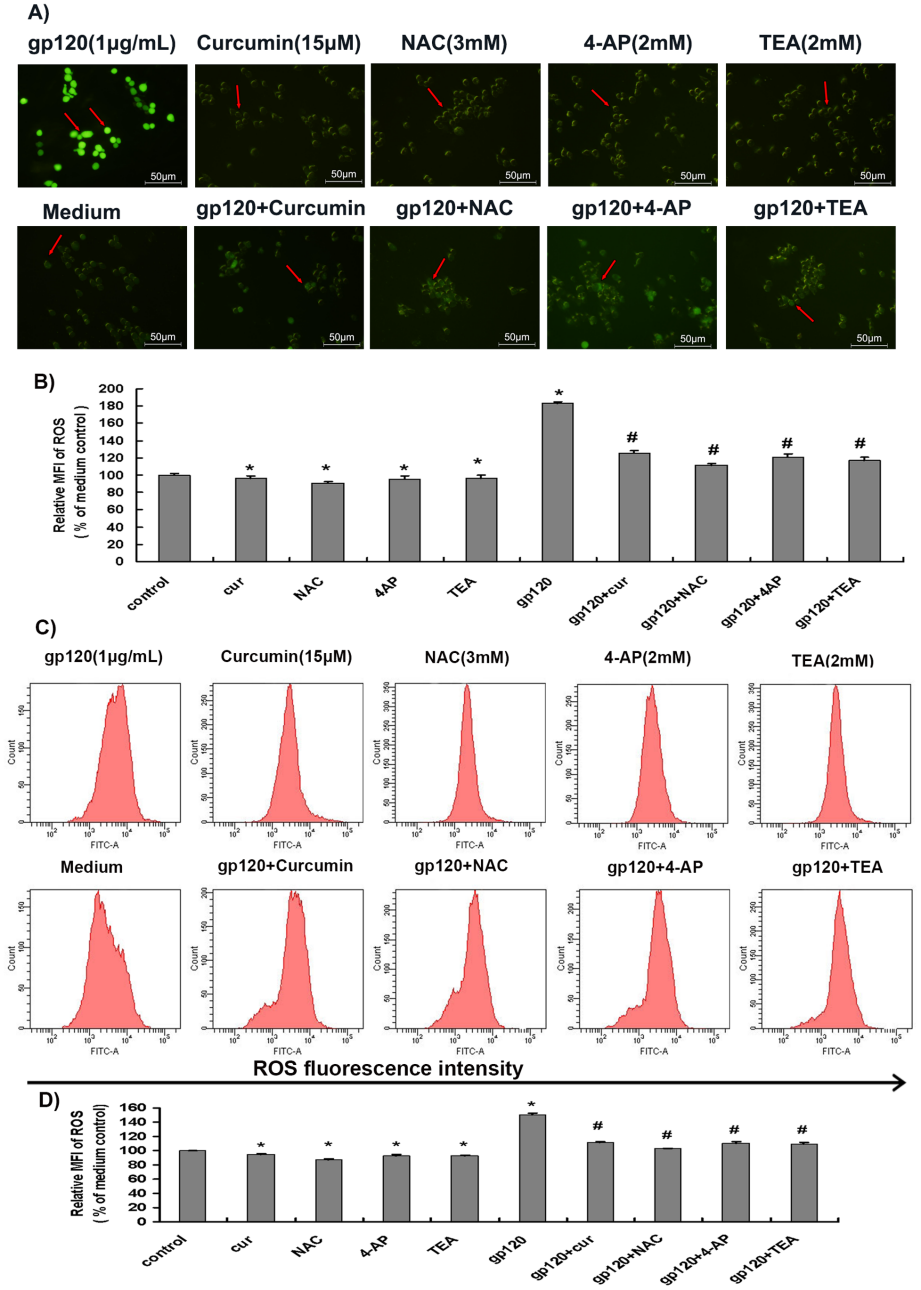
Curcumin, NAC, TEA and 4-AP alleviated HIV-1 gp120 V3 loop-induced ROS increase in microglia. After pre-incubation of microglia with curcumin (15 µM), NAC (3 mM), TEA (2 mM) or 4-AP (2 mM) for 2 h, cells were treated with HIV-1 gp120 V3 loop (1 µg/mL) for 1 h, and then subjected to ROS measurement using fluorescence microscope and FACS. A) and B): ROS production in microglia was determined using fluorescence microscope. The arrows indicated ROS-positive microglial cells. C) and D): ROS production in microglia was determined using FACS. Six independent experiments were performed. Each bar graph shows changes of ROS MFI calculating from comparison of experimental group versus medium control. *, *P*<0.05 vs. control, n = 6; #, *P*<0.05 vs. HIV-1 gp120 V3 loop alone, n = 6. Cur, curcumin.

The generation of ROS in microglial cells is directly associated with microglial viability [2]. We then investigated whether HIV-1 gp120-induced reduction of microglial viability was associated with production of ROS in microglial cells. We used a fluorescence microscopy to measure ROS levels in microglia treated or untreated with HIV-1 gp120 V3 loop (1 µg/mL), curcumin (15 µM), NAC (3 mM), 4-AP (2 mM), TEA (2 mM), or their combinations. We found that ROS levels in microglial cells treated with HIV-1 gp120 V3 loop was increased to 182.6±2.6% when compared to the medium control (Fig. 2A, 2B). In contrast, ROS level in cells treated with curcumin (15 µM), NAC (3 mM), 4-AP (2 mM), TEA (2 mM) was decreased to 96.0±2.9%, 90.1±2.5%, 95.7±3.8% and 95.9±3.9%, respectively, when compared to the medium control (Fig. 2A, 2B). ROS levels in the groups treated with HIV-1 gp120 V3 loop plus curcumin, HIV-1 gp120 V3 loop plus NAC, HIV-1 gp120 V3 loop plus 4-AP and HIV-1 gp120 V3 loop plus TEA decreased to 125.8±2.8%, 110.9±2.4%, 120.3±4.0%, and 116.7±3.7%, respectively, when compared to that of medium control (Fig. 2A, 2B). Statistical analysis showed that gp120 V3 loop significantly increased production of ROS in microglial cells (*p*<0.05), whereas curcumin, NAC, 4-AP, and TEA significantly inhibited production of ROS in microglia. Importantly, gp120-mediated ROS production was significantly alleviated by curcumin, NAC, 4-AP, or TEA (*p*<0.05) (Fig. 2A, 2B).

The data obtained by fluorescence microscopy analysis as described above were confirmed by FACS. The relative ROS levels in the groups treated with curcumin (15 µM), NAC (3 mM), 4-AP (2 mM), and TEA (2 mM) were 95.1±0.4%, 87.8±1.0%, 93.2±1.3%, and 92.4±1.8%, respectively, when compared to the medium control (Fig. 2C, 2D), whereas HIV-1 gp120 V3 loop increased intracellular ROS production to 150.8±2.0% of that in the medium control. Statistical analysis of these FACS data also showed that gp120 V3 loop significantly increased production of ROS in microglial cells (*p*<0.05), whereas curcumin, NAC, 4-AP, and TEA significantly inhibited production of ROS in microglia (*p*<0.05)(Fig. 2C, 2D). FACS data also showed that curcumin, NAC, 4-AP, and TEA significantly inhibited gp120-mediated production of ROS in microglia, as gp120 V3 loop plus curcumin, gp120 V3 loop plus NAC, gp120 V3 loop plus 4-AP, and HIV-1 gp120 V3 loop plus TEA significantly decreased ROS levels to 112.1±0.1%, 103.0±0.4%, 110.2±2.8%, and 109.0±2.5%, respectively, when compared to that of medium control (Fig. 2C, 2D). These values are significantly lower than that in gp120 V3 loop alone (*p*<0.05). Thus, both fluorescence microscopy and FACS analyses demonstrate that HIV-1 gp120 induces ROS in microglial cells, and this effect is alleviated by curcumin, NAC, 4-AP, or TEA.

### Curcumin Reduced HIV-1 gp120-triggered Production of Inflammatory Mediators in Microglia

**Figure 3:**
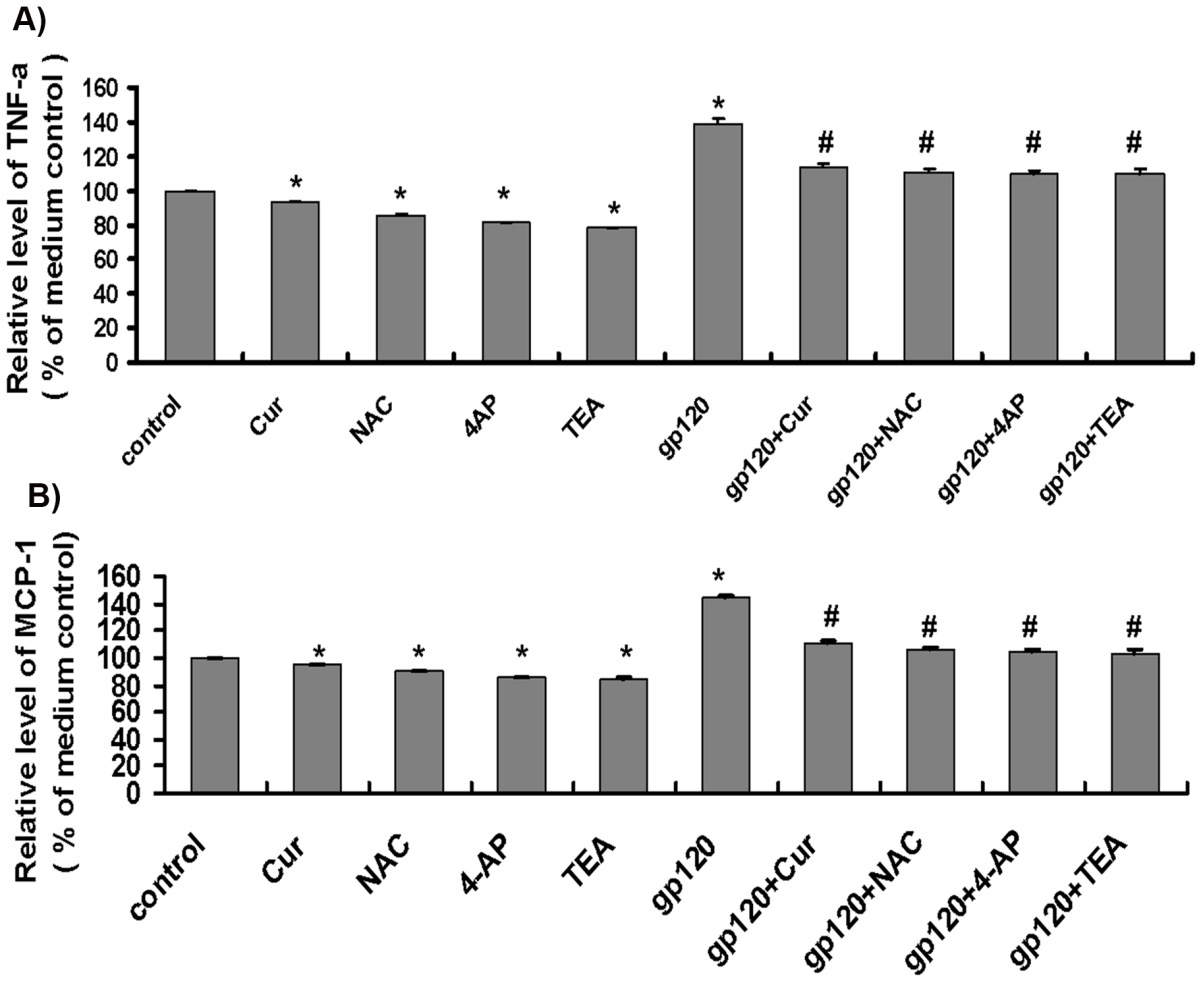
Curcumin, NAC, 4-AP and TEA reduced HIV-1 gp120 V3 loop-induced TNF-α and MCP-1 mRNA expression in microglia. After pre-incubation of microglia with curcumin (15 µM), NAC (3 mM), TEA (2 mM) or 4-AP (2 mM) for 2 h, cells were treated with HIV-1 gp120 V3 loop (1 µg/mL) for 3 h. Cells were then subjected to RNA extraction for measuring TNF-α and MCP-1 mRNA expression using a qPCR assay. A) and B): TNF-α mRNA and MCP-1 mRNA expression, respectively. *, *p*<0.05 vs. control, n = 6; #, *p*<0.05 vs. HIV-1 gp120 V3 loop alone. Experiments were repeated 6 times (n = 6). Cur, curcumin.

Microglia are the resident macrophages of the brain and spinal cord. These cells are constantly scavenging the CNS for infectious agents and damaged neurons. Microglial cells are able to destroy invading pathogens through physical contact (phagocytosis) and through producing a variety of cytotoxic substances such as inflammatory factors. We then used qPCR to study inflammatory responses of microglia to gp120 treatment. We found that gp120 significantly enhanced both TNF-α and MCP-1 mRNA levels in microglia. When compared to medium control (100%), gp120 significantly increased TNF-α and MCP-1 to 139.1±3.1% and 144.1±2.1%, respectively (*p*<0.05) (Fig. 3A, 3B). In contrast, curcumin (15 µM), NAC (3 mM), 4-AP (2 mM), and TEA (2 mM) suppressed both TNF-α and MCP-1 mRNA levels in microglia (Fig. 3A, 3B). Compared to medium control, curcumin, NAC, 4-AP, and TEA reduced TNF-α mRNA expression to 93.2±0.4%, 85.9±0.3%, 81.3±0.4%, and 78.4±0.4%, and MCP-1 mRNA expression to 94.6±0.5%, 89.9±0.5%, 85.9±0.4% and 84.4±0.5%, respectively (Fig. 3A, 3B). Importantly, gp120-enhanced production of TNF-α and MCP-1 was significantly alleviated by curcumin, NAC, 4-AP, or TEA, as TNF-α mRNA levels in gp120 V3 loop plus curcumin, gp120 V3 loop plus NAC, gp120 V3 loop plus 4-AP, and gp120 V3 loop plus TEA groups decreased to 113.7±2.5%, 110.5±1.8%, 109.5±1.9% and 109.4±3.2%, and MCP-1 mRNA levels in these groups decreased to 110.4±2.1%, 105.6±1.8%, 104.6±1.9% and 103.0±2.5%, respectively (Fig. 3A, 3B). Levels of TNF-α and MCP-1 mRNA expression in all groups of curcumin, NAC, 4-AP, and TEA were significantly lower than that in gp120 treatment (*p*<0.05), indicating that curcumin, NAC, 4-AP and TEA attenuate gp120 activity in induction of inflammatory factors in microglial cells.

### Curcumin Ameliorated HIV-1 gp120 V3 Loop-mediated Apoptosis of Cortical Neurons

**Figure 4:**
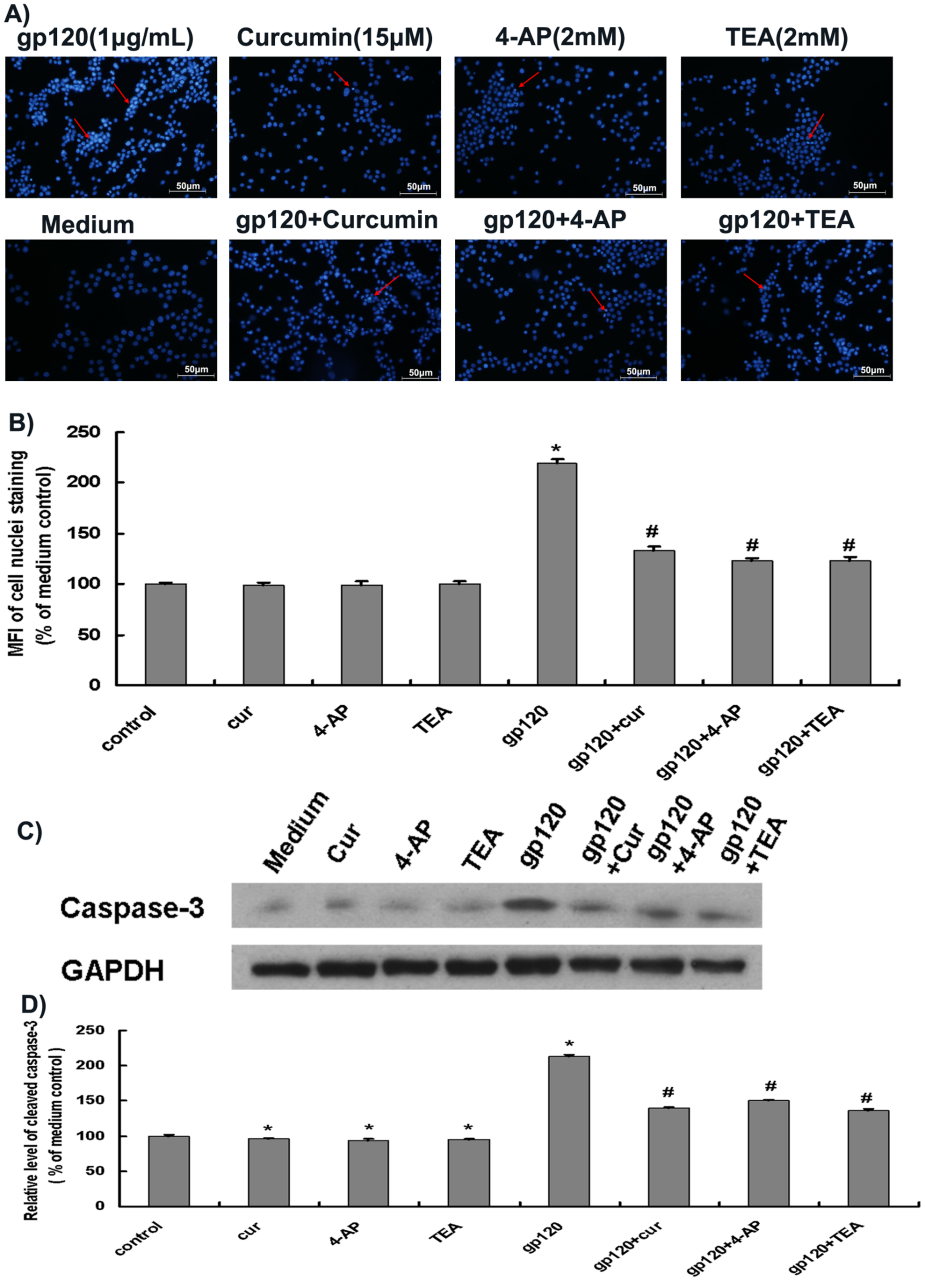
Curcumin, 4-AP and TEA reduced HIV-1 gp120 V3 loop- induced apoptosis of primary rat cortical neurons. Primary rat cortical neurons that had been cultured for 7 days were treated with corresponding conditioned media for 24 h and then subjected to apoptosis analysis. A) and B): Apoptosis of neurons was determined by Hoechst 33342 staining. Each bar shows % of MFI of Hoechst 33342-stained cortical neuron nuclei versus medium control or gp120 treatment alone. C) and D): Apoptosis of neurons was determined by western blotting analysis of cleaved caspase-3 levels. Each bar shows % of cleaved caspase-3 level vs medium control or gp120 treatment alone. *, *p*<0.05 vs. control, n = 6; #, *p*<0.05 vs. HIV-1 gp120 loop alone, n = 6. The arrows indicate the neuronal nuclei in each group. Cur, curcumin.

Apoptosis, an active process of cell death characterized by cell shrinkage, chromatin aggregation with genomic fragmentation and nuclear pyknosis, is an important feature of HIV-1-associated central neurological dysfunction. Apoptotic neurons have been observed in the CNS of HIV-1-infected individuals [2]. We then studied the effects of HIV-1 gp120 and curcumin on apoptosis of primary neurons. Hoechst 33342 staining showed that the nuclei of primary rat cortical neurons in the medium control were uniform and bluish (Fig. 4A). In contrast, the nuclei of primary rat cortical neurons treated with HIV-1 gp120 V3 loop were dense and in bright blue (Fig. 4A). The MFI of cells treated with gp120 was 220.1±3.6% when compared to that in medium control, whereas MFI of cells treated with curcumin (15 µM), 4-AP (2 mM), or TEA (2 mM) were 99.0±2.7%, 98.4±3.9%, and 99.8±2.7%, respectively (Fig. 4B). The MFI of neurons in each of these groups was significantly lower that in gp120 group (*p*<0.05) (Fig. 4B). In addition, numbers of bright blue nuclei in gp120 V3 loop plus curcumin, gp120 V3 loop plus 4-AP, and gp120 V3 loop plus TEA treatment groups were decreased, and MFI of the nuclei in these groups were 133.0±4.4%, 123.4±2.2%, and 122.8±3.8%, respectively, when compared to that in medium control (Fig. 4A, 4B). Statistical analysis showed that curcumin, 4-AP, and TEA significantly alleviated HIV-1 gp120 V3 loop-induced apoptosis of neurons (Fig. 4B).

Next, we investigated whether HIV-1 gp120 and curcumin affected neuronal apoptosis through the caspase*-*dependent pathway. Compared with the medium control, gp120 significantly increased caspase-3 expression to 213.3±2.8% (*p*<0.05), whereas curcumin, 4-AP, and TEA decreased caspase-3 expression to 96.1±1.5%, 94.0±2.5%, and 94.4±1.6%, respectively (Fig. 4C, 4D). The effects by each of these reagents were also significant (*p*<0.05)(Fig. 4D). The relative cleaved caspase-3 levels in groups of gp120 V3 loop plus curcumin, gp120 V3 loop plus 4-AP, and gp120 V3 loop plus TEA were 139.3±2.0%, 150.3±1.8% and 136.0±2.6%, respectively, when compared to that in the medium control (Fig. 4C, 4D). These values are significantly lower than that in gp120 V3 loop alone (Fig. 4D). These results indicate that HIV-1 gp120 V3 loop induces apoptosis of primary rat cortical neurons through activation of the caspase-dependent pathway, and the effect is alleviated by curcumin, 4-AP and TEA.

### Curcumin Attenuated HIV-1 gp120 V3 Loop-mediated Increase of the Delayed Rectification and the Transient Outward K+ Current

**Figure 5:**
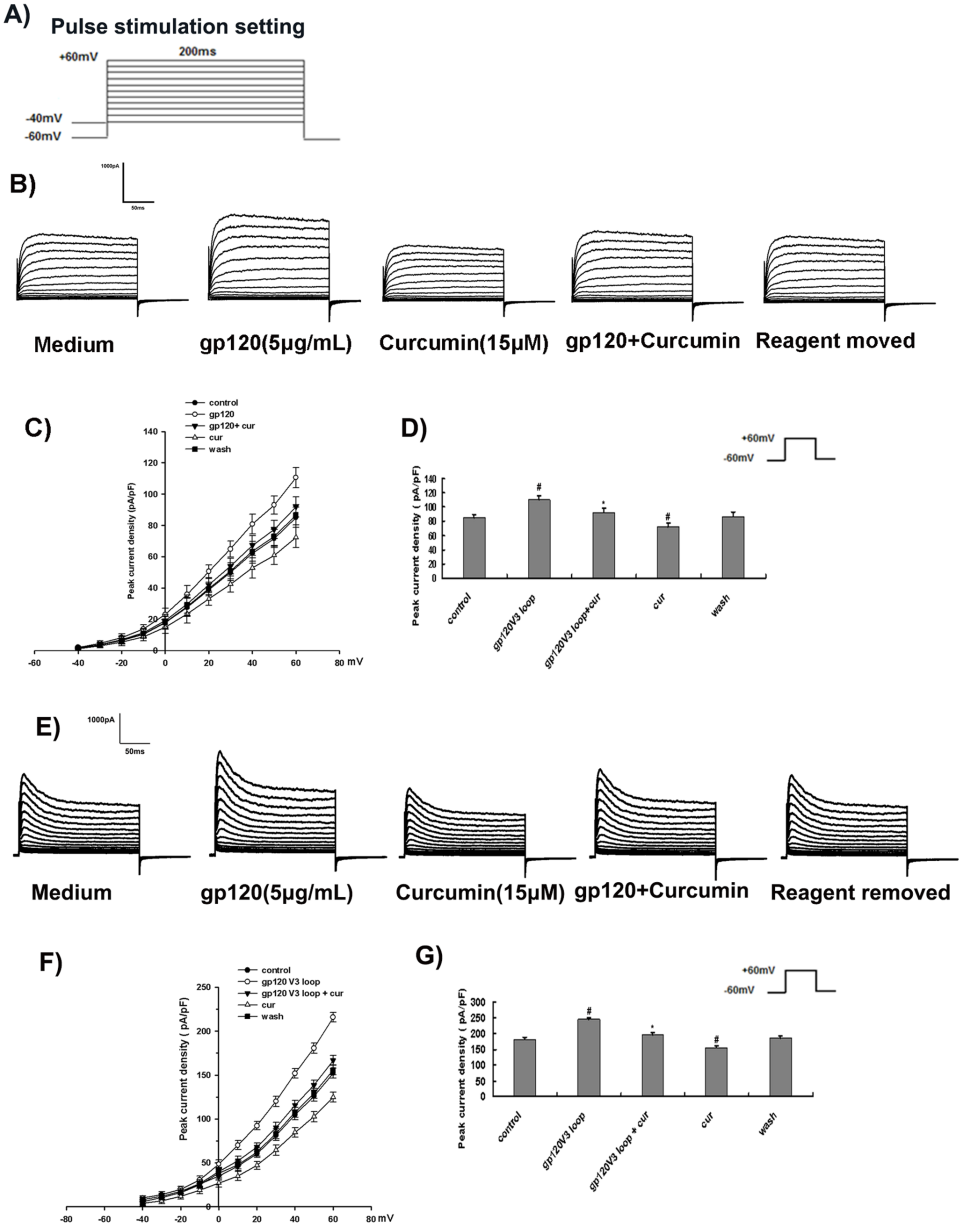
Curcumin attenuated HIV-1 gp120 V3 loop-induced increase in the delayed rectifier K^+^ current and the transient outward K^+^ current. Whole-cell patch clamp recordings of primary rat cortical neuronal bioactivity in response to gp120 (5 µg/mL) treatment with or without curcumin (15 µM) were performed in 35-mm culture dishes on an inverted Olympus microscope stage using an Axopatch 200B amplifier. Whole-cell delayed rectifier K^+^ current and transient outward K^+^ current were induced through voltage steps ranging from −60 mV to +60 mV in 10-mV increments. A): Schematic representation of the pulse stimulation setting for both recordings of delayed rectifier K^+^ current and transient outward K^+^ current. B): A recording of delayed rectifier potassium current in medium (control), cells treated with gp120 (5 µg/mL), curcumin (15 µM),gp120 (5 µg/mL) plus curcumin (15 µM), or post-washing. C): I-V curves of delayed rectifier K^+^ current densities in response to gp120 (5 µg/mL) treatment with or without curcumin (15 µM). D): Pooled whole-cell recording data of the delayed rectifier K^+^ current from 6 separated experiments. E): A recording of transient outward K^+^ current in medium (control), cells treated with gp120 (5 µg/mL), curcumin (15 µM),gp120 (5 µg/mL) plus curcumin (15 µM), or post-washing. F: I–V curves of transient outward K^+^ current densities in response to gp120 (5 µg/mL) treatment with or without curcumin (15 µM). G: Pooled whole-cell recording data of the transient outward K^+^ current from 6 separated experiments. Each value represents the mean ± SEM. #, *p*<0.05 vs. control; *, *p*<0.05 vs. HIV-1 gp120 V3 loop alone; n = 6.

To address whether HIV-1 gp120 directly affects neuron activity leading to neurotoxicity, we treated primary rat cortical neurons with gp120 to study its effects on delayed rectification and transient outward K^+^ currents. Under voltage clamp conditions, cells were maintained at −60 mV, and depolarizing positive pulses for up to −20 mV elicited outward currents (Fig. 5A). We recorded the K^+^ current as the background. The current amplitudes were increased with stronger depolarization (Fig. 5C, 5F). When the membrane potential was depolarized to +60 mV, the average peak outward current densities in the control were 85.1±4.0 pA/pF (delayed rectifier K^+^ current; Fig. 5B, 5D) and 152.1±5.1 pA/pF (transient outward K^+^ current; Fig. 5E, 5G). When HIV-1 gp120 V3 loop was added to the perfusion solution, the delayed rectifier and transient outward K^+^ current increased to 110.6±5.1 pA/pF and 215.9±4.6 pA/pF, respectively (Fig. 5D, 5G). The difference of the delayed rectifier or transient outward K^+^ current between the control and HIV-1 gp120 V3 loop groups was statistically significant (*p*<0.05, n = 6; Fig. 5D, 5G).

Next, we added gp120 V3 loop plus curcumin to the perfusion solution and found that the delayed rectifier and transient outward K^+^ currents were decreased compared to HIV-1 gp120 V3 loop alone. The average peaks of the delayed rectifier and transient outward K^+^ current densities at +60 mV were 91.9±6.4 pA/pF and 166.7±5.9 pA/pF, respectively (Fig. 5B, 5E). The difference of these values between HIV-1 gp120 V3 loop and HIV-1 gp120 V3 loop plus curcumin groups was statistically significant (*p*<0.05, n = 6; Fig. 5D, 5G). When we added curcumin alone to the perfusion solution, the delayed rectifier and transient outward K^+^ current at +60 mV decreased to 72.3±5.8 pA/pF and 124.8±5.5 pA/pF, respectively (Fig. 5B, 5D, 5E, 5G). The current amplitude returned to nearly the medium control level after washing of cells to remove the reagents (Fig. 5B, 5E). The average peak outward current densities at +60 mV were 86.8±6.5 pA/pF (delayed rectifier K^+^ current; Fig. 5D) and 155.8±5.9 pA/pF (transient outward K^+^ current; Fig. 5G). These data indicate that the HIV-1 gp120 V3 loop may damage neurons by increasing both the delayed rectification and the transient outward K^+^ current, and these effects may be attenuated by curcumin, resulting in protection of neuronal injury against gp120 neurotoxicity.

## Discussion

Curcumin is a natural component of the rhizome of Turmeric (Curcuma longa) and has been used traditionally for centuries by Chinese medicine. Curcumin has an outstanding safety profile and has shown a number of pleiotropic actions including anti-inflammatory, antioxidant, anti-tumor properties and anti-protein-aggregate activities [16], [17]. Curcumin also has neuronal protections [18]. Because of its long history of use, safety, pluripotency, and inexpensive cost, curcumin has a great potential role in the prevention and treatment of neurological disorders for which current therapeutics are less than optimal [16]. For example, curcumin is a promising agent in the treatment and prevention of Alzheimer’s disease (AD), a progressive neurodegenerative disease [19]. We therefore hypothesized that curcumin might have a protective effect against HIV-1-assocated neurological disorders. We found that HIV-1 gp120 profoundly induced N9 microglial cells to produce ROS, TNF-α and MCP-1 (Fig. 2, and Fig. 3), eventually might lead to decreased viability of microglial cells (Fig. 1). Curcumin exerted a powerful inhibitory effect against HIV-1 gp120-induced neuronal damage, reducing the production of ROS, TNF-α and MCP-1 by N9 microglial cells. In fact, curcumin at 15 µM exhibited the same suppressive activity in decreasing ROS levels in gp120-treated microglial cells as that of NAC at 3 mM, an optimal concentration of the potent antioxidant (Fig. 2), suggesting that curcumin is also an potent antioxidant. Induction of ROS causes inflammation and the reverse sequence of this event is also true. Curcumin at 15 µM also suppressed production of inflammatory factors including TNF-α and MCP-1 in gp120-treated N9 microglial cells to the same extent as NAC treatment at 3 mM. Blocking voltage-gated potassium channels decreased ROS level and cytokine mRNA production in gp120-treated microglial cells as well. This finding indicates that the voltage-gated potassium channels are related to the ROS and cytokine production induced by the HIV-1 gp120 V3 loop. Curcumin may play a microglial protective role by reducing voltage-gated potassium channel currents. Curcumin is able to cross the blood-brain barrier (BBB) to provide direct neuroprotection in AD models [20]. We also have demonstrated that curcumin alleviates HIV-1-gp120- induced memory impairment and hippocampal neuron injury in rats [13], [21]. Our data together with these reports indicate that curcumin represents a potential component for preventive and therapeutic interventions of HIV-1-associated neurological disorders.

HIV-1 causes neuronal injury, cell loss and dysfunction in the CNS through soluble virus proteins rather than productive virus infection, because there is no evidence that HIV-1 directly infects neurons. HIV-1 gp120 protein has been shown to directly interact with neurons, leading to neuronal apoptosis [3]. In agreement with these results, we found that HIV-1 gp120 directly induced apoptosis of primary rat cortical neurons. Neuronal apoptosis is known to be accompanied by a concomitant increase in Kv channel expression in the plasma membrane [22], [23], [24]. Such an increase in Kv expression is believed to facilitate an increased K^+^ efflux, leading to a loss of cytosolic K^+^. This process is accompanied by Cl^−^ outﬂow and water efflux, eventually leading to cell shrinkage and the induction of apoptotic signals [25]. We found that HIV-1 gp120 significantly increased both the delayed rectification and the transient outward K^+^ current in primary rat cortical neurons (Fig. 5). These effects were attenuated by curcumin, resulting in protection of neuronal injury against gp120 neurotoxicity (Fig. 4 and Fig. 5). An increase in the Kv current and in apoptosis has been observed in beta-amyloid-treated cortical neurons [26]. Both TEA and 4-AP have shown their activities in attenuation of beta-amyloid-induced neuronal death through suppressing K^+^ efflux [27], [28] ^.^ Curcumin also alleviated HIV-1 gp120-mediated increase in the delayed rectification and the transient outward K^+^ current. Thus, curcumin inhibits gp120-mediated neuronal apoptosis, most likely through inhibition of HIV-1 gp120-induced elevation of the delayed rectification and the transient outward K^+^ current.

In summary, HIV-1 gp120 increases ROS, TNF-α and MCP-1 production in microglia, and induces cortical neuron apoptosis by affecting the delayed rectification and transient outward K^+^ channel current. Curcumin reduces production of ROS and inflammatory mediators in HIV-1-gp120-stimulated microglia, and protects cortical neurons against HIV-1-mediated apoptosis, most likely through inhibition of HIV-1 gp120-induced elevation of the delayed rectification and transient outward K^+^ current.
